# Late relapse in acute myeloid leukemia (AML): clonal evolution or therapy-related leukemia?

**DOI:** 10.1038/s41408-019-0170-3

**Published:** 2019-01-16

**Authors:** Musa Yilmaz, Feng Wang, Sanam Loghavi, Carlos Bueso-Ramos, Curtis Gumbs, Latasha Little, Xingzhi Song, Jianhua Zhang, Tapan Kadia, Gautam Borthakur, Elias Jabbour, Naveen Pemmaraju, Nicholas Short, Guillermo Garcia-Manero, Zeev Estrov, Hagop Kantarjian, Andrew Futreal, Koichi Takahashi, Farhad Ravandi

**Affiliations:** 1 Department of Leukemia, The University of Texas MD Anderson Cancer Center, Houston, TX USA; 2 Department of Genomics, The University of Texas MD Anderson Cancer Center, Houston, TX USA; 3 Department of Hematopathology, The University of Texas MD Anderson Cancer Center, Houston, TX USA

## Abstract

Late relapse, defined as relapse arising after at least 5 years of remission, is rare and occurs in 1–3% of patients with acute myeloid leukemia (AML). The underlying mechanisms of late relapse remain poorly understood. We identified patients with AML who achieved remission with standard induction chemotherapy and relapsed after at least five years of remission (*n* = 15). Whole exome sequencing was performed in available bone marrow samples obtained at diagnosis (*n* = 10), remission (*n* = 6), and first relapse (*n* = 10). A total of 41 driver mutations were identified, of which 11 were primary tumor-specific, 17 relapse-specific, and 13 shared (detected both in primary and relapsed tumor samples). We demonstrated that 12 of 13 shared mutations were in epigenetic modifier and spliceosome genes. Longitudinal genomic characterization revealed that in eight of 10 patients the founder leukemic clone persisted after chemotherapy and established the basis of relapse years later. Understanding the mechanisms of such quiescence in leukemic cells may help designing future strategies aimed at increasing remission duration in patients with AML.

## Introduction

A majority of the patients with newly diagnosed acute myeloid leukemia (AML) achieve complete remission (CR) with intensive chemotherapy. However, approximately two-thirds of patients relapse after frontline therapy and most relapses occur within the first 18 months. Late relapses, arbitrarily defined as any relapse occurring after 5 years of remission, have been reported in 1–3% of the patients with AML^1–3^.

The underlying pathophysiology of late relapse remains elusive. Most cases of late relapse are associated with a normal karyotype^2^ or lack typical cytogenetics abnormalities observed in therapy-related AML^4^. This raises the question whether these cases are true late relapses or they are associated with prior exposure to chemotherapy. Indeed, if these are true late relapses, understanding such prolonged quiescence of AML may provide further insights into developing superior strategies for treating these patients.

In a recent study, we performed whole exome sequencing (WES) in the primary tumor-relapse bone marrow pairs from one patient with AML who relapsed after 19 years of remission^5^. We showed that the founding clone persisted after initial chemotherapy and relapsed with additional acquired mutations. In current study, our aim is to identify all patients who relapsed after 5 years of remision and perform WES in primary tumor-relapse pairs to characterize molecular aberrations causing late relapses.

## Methods

### Patients

**Fig. 1:**
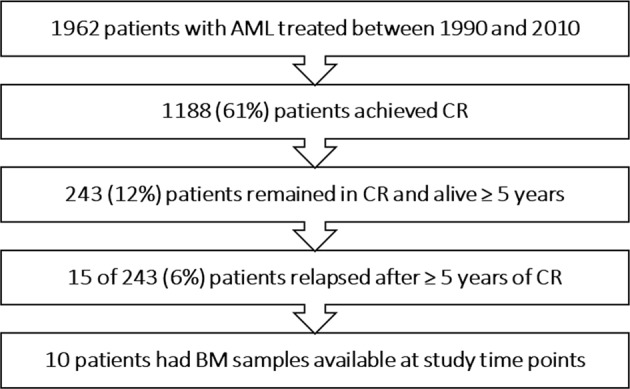
Patient selection *AML* acute myeloid leukemia, *CR* complete remission, *BM* bone marrow

We analyzed patients with newly diagnosed AML (excluding acute promyelocytic leukemia) who received induction chemotherapy between 1990 and 2010 at MD Anderson Cancer Center, and identified 15 patients who met our criteria of late relapse (Fig. 1). Bone marrow (BM) samples collected from patients with late relapse (*n* = 15) at diagnosis, CR, and at first relapse were analyzed by WES. Patients were identified with unified patient numbers (UPN) from UPN1 to UPN15. We performed WES at specified time points for 10 patients (UPN1,3,5,6,7,9,10,12,14, and 15). WES was not attempted for five patients due to lack of either baseline or relapse BM sample (UPN2,4,8,11, and 13). WES was performed at the specified time points for all remaining ten patients (total three WES per patient), and UPN9 had an additional diagnostic sample (leukemia cutis) sequenced. Remission BM samples (collected immediately after achieving remission) were available for six of 10 patients (UPN3,6,7,12,14, and 15). This study was approved by the institutional review board of MD Anderson Cancer Center.

### Whole exome sequencing

Genomic DNA was extracted from the patient’s samples and Agilent SureSelect All Exon V5 was used for exome capture hybrid. Sequencing with 75 base pair paired-end read was performed with Illumina HiSeq 2000 sequencer. Sequencing data were aligned to the hg19 human genome reference using Burrows-Wheeler Aligner (BWA)^6^ followed by indel realignment, mark duplication, and base recaliberation using GATK best practices tools (https://www.broadinstitute.org/gatk/guide/best-practices?bpm = DNAseq). The resulting BAM files were preprocessed and small insertions/deletions and base substitutions were called using Mutect^7^ and Pindel^8^, respectively, against the unmatched normal sample. Non-hematopoietic germline control DNA was not used due to sample unavailability. Further details regarding variant calling and filtering methods can be found in the methods section of Supplementary Appendix.

### Cytogenetic analysis

Cytogenetic testing was conducted on unstimulated BM cells after culture (24–72 h), and G-banding analysis was performed according to standard techniques. European leukemia network (ELN) cytogenetic risk classification was used to stratify patients’ risk factors^9^.

### Statistics

The statistical analysis was performed by using SPSS software (Version 23.0. Armonk, NY: IBM Corp). Mann-Whitney U, Kruskal-Wallis, or Chi-square test was used to evaluate associations between variables. Categorical variables were reported as percentages and counts. Continuous variables were reported as median (interquartile range (IQR)) or mean. All *P* values are two-tailed and a *P* value <0.05 was considered as statistically significant. Response to induction or salvage chemotherapy was defined according to revised International Working Group^10^.

## Results

### Clinical characteristics

Among 1962 patients with AML who received induction chemotherapy, 1188 (61%) achieved remission; 243 (20%) were alive and in remission for at least 5 years and 15 (6%) of these relapsed during follow-up. Overall, 703 patients relapsed, and late relapses constituted 2% of all relapses (15 of 703).

At diagnosis, the median age was 58 years (range 17–75); 13 patients (80%) had intermediate-risk, one favorable-risk (7%), and one poor-risk karyotype (7%) (Supplementary Table 1). Eleven of the 15 patients (73%) had a normal karyotype. One patient (UPN11) had isolated extramedullary myeloid sarcoma both at diagnosis and relapse. Thirteen patients (87%) were induced with various intensive chemotherapy regimens, and two received low-intensity regimens. Only one patient received allogeneic stem cell transplantation (allo-SCT) at CR1 (UPN3). The overall median CR1 duration was 7.4 years (range 5.5–24.1).

At relapse, nine of 15 (60%) patients had normal karyotype, and six (40%) developed completely new (*n* = 5) or additional (*n* = 1) chromosome abnormalities (Supplementary Table 2). All patients received salvage chemotherapy at relapse; seven patients were treated with intensive regimens, and eight received low-intensity regimens. Overall response rate was 80% (12 of 15), with CR and CRi rates of 60% (*n* = 9) and 20% (*n* = 3), respectively.

### Clonal evolution

**Fig. 2:**
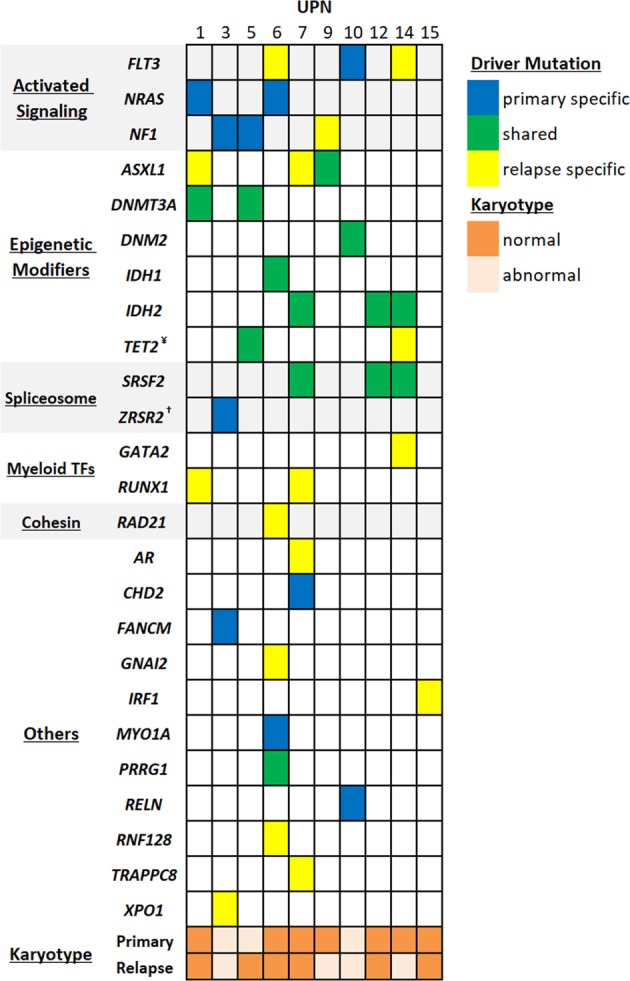
Karyotype and driver mutations in 10 primary-relapse tumor pairs. ^¥^UPN5 had two TET2 mutations; shared (c.3347delT:p.111 fs) and relapse-specific (c.C4609T:p.R1537X) [not shown in figure]. ^†^UPN3 had two primary-specific ZRSR2 mutations; c.865delG:p.G289fs and c.1239delG:p.K413fs

The mean WES coverage was 154x for 27 sequenced samples. A total of 41 driver mutations were identified in 10 patients, of which 11 were primary tumor-specific, and 17 relapse-specific (Fig. 2). Thirteen driver mutations were detected both in primary and relapsed tumor samples (shared). Twelve of the 13 (92%) shared driver mutations involved epigenetic modifier or spliceosome-complex genes. Twenty-five of 41 driver mutations (60%) were nonsynonymous single nucleotide variation (SNV), and 16 (40%) were frameshift insertions and deletions (indels).

**Fig. 3:**
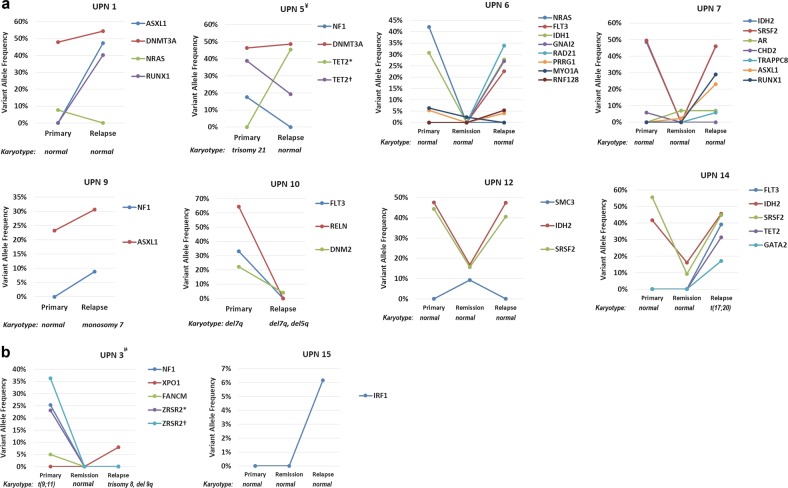
Karyotype and VAF of driver mutations in primary tumor (*n* = 10), remission (*n* = 6) and relapse (*n* = 10) tumors. **a** The mutations detected in the primary and relapse tumor samples from eight patients (UPN1,5,6,7,9,10,12 and 14) with AML: the founding clone in the primary tumor gained relapse-specific mutations or cytogenetic abnormalities and evolved into a relapse clone. **b** The mutations detected in the primary and relapse tumor samples from two patients (UPN3 and 15) with AML: the founding clone detected at relapse was different from the founding clone at diagnosis (the relationship between mutations in the primary and relapse tumors are shown by lines linking them). Remisison bone marrow samples were available only for six patients VAF: variant allele frequency ^¥^UPN5: *p.R1537X, ^†^p.I1116fs ^µ^UPN3: *p.G289fs, ^†^p.K413fs

The generation of sequencing data allowed us to detect driver mutations and quantify variant allele frequencies (VAF) in 10 primary-relapse tumor pairs, permitting estimation of the size of tumor populations. We demonstrated that in eight cases (UPN1,5,6,7,9,10, 12, and 14) the founding clone in the primary tumor gained relapse-specific mutations and evolved into a relapse clone (Fig. 3a). Only in two cases (UPN3 and 15), the founding clone detected at relapse was different from the founding clone at diagnosis (Fig. 3b).

In five cases (UPN1, 5, 6, 7, and 10), some of the subclones in primary tumor were eradicated with chemotherapy and not detected at relapse (Fig. 3a). UPN6 harbored *IDH1*, *PRRG1, MYO1A*, and *NRAS* mutations at diagnosis, and lost *MYO1A* and *NRAS*, but gained four additional mutations, *FLT3, GNAI2, RAD21*, and *RNF128*, at relapse. UPN10 was the only case with no new mutation detected at relapse, in which karyotype analysis revealed clonal evolution. UPN10 had *DNM2, RELN, and FLT3* mutations and del7q at diagnosis, and DNM2 was the only persistent mutation detected at relapse (VAF 4%). The del7q also persisted in the relapse tumor, and additionally, it harbored a new karyotype abnormality, del5q, which was not detected 6 years ago at diagnosis.

UPN3 and UPN15 followed relapsing patterns that were distinct from other cases (Fig. 3b). At diagnosis, UPN3 harbored *ZRSR2, FANCM*, and *NF1* mutations and t(9;11). However, none of these alterations were detected 12 years later at relapse. Instead, the relapsing disease had *XPO1* mutation and new karyotype abnormalities, such as trisomy 8 and del9q. UPN3 was the only patient who received allo-SCT in CR1, and at relapse chimerism analysis showed 100% donor cells. UPN15 revealed a relapse-specific IRF1 mutation (low VAF, 6%) and no other mutations. Karyotype at relapse also remained normal.

The median time to remission was 33 days (range 20–44), and WES was performed successfully in six patients with available samples obtained immediately after remission (UPN3, 6, 7, 12, 14, and 15) (Fig. 3). In total, eight driver mutations detected at remission: *IDH2* and *SRSF2* in both UPN12 and UPN14, *ASXL1* and *AR* in UPN7, *MYO1A* in UPN6, and *SMC3* in UPN12. Among six cases with a total of 16 driver mutations at baseline, three had persistent mutations in remission (total five driver mutations). Although numbers were small to draw a definitive conclusion, mutations detected at remission were more likely to persist and be detected at relapse. Of the eight driver mutations detected at remission, six were also detected at relapse.

UPN9 had BM and skin involvement, and we sequenced both samples at diagnosis. While bone marrow sample revealed *ASXL1* mutation, WES was not able to capture any driver mutation from skin sample.

## Discussion

Late relapse is uncommon in patients with AML, and constitutes ~2% of all relapses. WES in 10 primary-relapse tumor pairs revealed that in eight cases the founder clone persisted after frontline therapy and relapsed after a median of 7 years. We identified loss of primary tumor subclones in five of eight cases, suggesting that some of the subclones were eliminated with chemotherapy. In all cases except one, relapsing tumor acquired at least one relapse-specific mutation.

UPN3 relapsed after allo-SCT with chimerism analysis showing 100% donor cells. The relapsed disease harbored completely new driver mutations and karyotype abnormalities. These findings suggest that “relapse” may be a new primary and the clone may potentially have originated from the graft itself.

UPN15 had no molecular aberration at diagnosis, and IRF1 was the only mutation detected at relapse. Karyotype remained normal during all time points. One possibility is that our sequencing platform was not sensitive enough to detect mutations in this case. Another possibility is that the initiating driver of AML in UPN15 could be a cryptic fusion gene, such as NUP98-NSD1 or others, which were not analyzed in our study.

In a study by Ding et al., whole genome sequencing was performed in baseline and relapse BM samples of eight patients with AML (median time to relapse 351 days)^11^. The authors demonstrated that in some cases the founding clone survived initial chemotherapy, and gained additional mutations at relapse. With a long period of quiescence, AML patients with late relapse seem to follow similar clonal evolution pattern.

Clonal hematopoiesis (CH) is defined as the expansion of one lineage of cells, or a clone, at a rate disproportionately greater than other cell lineages. CH is a common, age-depended state, and is associated with increased risk of subsequent leukemia. Accumulating evidence suggests that primary mutations in epigenetic modifiers that occurred in hematopoietic stem cells lead to clonal hematopoietic expansion. Most common epigenetic modifiers associated with CH are *DNMT3A*, *TET2*, and *ASXL1*^12^. Although uncommon, mutations in *IDH* gene has also been identified in CH studies^13,14^. In a recent study, BM samples of patients with newly diagnosed AML were sequenced at diagnosis and remission^15^. With a median follow-up of 3.3 years, it was found that some of the CH-associated mutations (*DNMT3A, TET2, and ASXL1)* were persistent in 90 of 130 patients (68%) during remission, and their persistence was not associated with increased risk of relapse. The absence of sequencing at relapse prevents us to know whether these mutations persisted at relapse or not. However, in our study with a longer follow-up and sequencing performed at relapse, of eight patients who relapsed with the same founding clone, all harbored mutations in genes that are known to be associated with CH (Fig. 2). These mutations persisted for many years and manifested as part of relapsed disease along with newly acquired relapse-specific mutations. Overall, these findings suggest that the presence of CH-associated mutations at diagnosis may play role in relapse of patients with AML who remain in remission for 5 years or more.

The underlying pathophysiologic mechanisms of such long quiescence are unclear. Patients were, on average, 7 years older when they relapsed. Decreased immune surveillance against cancer with advanced age could be one of the contributing factors for late relapse. While older patients are by no means immunodeficient, they often do not respond to new or previously encountered antigens as immune system is altered with aging^16–21^. Understanding the influence of immunosenescence on cancer progression may lead to developing intervention strategies.

All patients in our study cohort received cytotoxic chemotherapy for induction and consolidation. DNA damage and selective pressure induced by chemotherapy may be associated with the development of secondary AML. However, nine of 15 (60%) patients relapsed with normal karyotype, and among six who relapsed with an abnormal karyotype, only three had cytogenetic changes (complex, deletion 5 or 7) that may be associated with prior cytotoxic chemotherapy. In a study, whole genome sequencing was performed in BM samples of 22 patients with therapy-related AML, and the results were compared with de-novo AML^22^. Patients with therapy-related AML were more likely to be associated with *TP53* and *ABC* family gene mutations and less likely to have *DNMT3A* and *NPM1*. In our study, none of the patients harbored *TP53* or *ABC* family gene mutations at relapse, and two relapsed with *DNMT3A*. The absence of common cytogenetic and molecular abnormalities seen in therapy-induced leukemia suggests other mechanisms for late relapse.

These results extend the findings of previous studies^11,23–25^, which described patterns of clonal evolution in leukemia patients, using sequencing techniques or copy number alterations. Our analysis provides complementary data on clonal evolution in late relapse AML. Longitudinal genomic characterization of patients with a late relapse of AML showed that in most cases the founder leukemic clone persisted after chemotherapy and established the basis of relapse years later. Understanding the mechanisms of such quiescence may assist in designing future strategies aimed at increasing remission duration in patients with AML.

## Supplementary information

Supplementary Appendix

## References

[1] Verma D (2010). Late relapses in acute myeloid leukemia: analysis of characteristics and outcome. Leuk. Lymphoma.

[2] Medeiros BC (2007). Characteristics and outcomes of acute myelogenous leukemia patients with very late relapse (>5 years). Leuk. Lymphoma.

[3] Kantarjian HM, Keating MJ, Walters RS, McCredie KB, Freireich EJ (1988). The characteristics and outcome of patients with late relapse acute myelogenous leukemia. J. Clin. Oncol..

[4] Pedersen-Bjergaard J, Andersen MT, Andersen MK (2007). Genetic pathways in the pathogenesis of therapy-related myelodysplasia and acute myeloid leukemia. Hematology Am. Soc. Hematol. Educ. Program.

[5] Takahashi K (2015). Clonal evolution of acute myeloid leukemia relapsed after 19 years of remission. Am. J. Hematol..

[6] Li H, Durbin R (2009). Fast and accurate short read alignment with Burrows-Wheeler transform. Bioinformatics.

[7] Cibulskis K (2013). Sensitive detection of somatic point mutations in impure and heterogeneous cancer samples. Nat. Biotechnol..

[8] Ye K, Schulz MH, Long Q, Apweiler R, Ning Z (2009). Pindel: a pattern growth approach to detect break points of large deletions and medium sized insertions from paired-end short reads. Bioinformatics.

[9] Dohner H (2017). Diagnosis and management of AML in adults: 2017 ELN recommendations from an international expert panel. Blood.

[10] Cheson BD (2003). Revised recommendations of the international working group for diagnosis, standardization of response criteria, treatment outcomes, and reporting standards for therapeutic trials in acute myeloid leukemia. J. Clin. Oncol..

[11] Ding L (2012). Clonal evolution in relapsed acute myeloid leukaemia revealed by whole-genome sequencing. Nature.

[12] Bowman RL, Busque L, Levine RL (2018). Clonal hematopoiesis and evolution to hematopoietic malignancies. Cell Stem Cell.

[13] Kunimoto H, Nakajima H (2017). Epigenetic dysregulation of hematopoietic stem cells and preleukemic state. Int. J. Hematol..

[14] Xie M (2014). Age-related mutations associated with clonal hematopoietic expansion and malignancies. Nat. Med..

[15] Jongen-Lavrencic M (2018). Molecular minimal residual disease in acute myeloid leukemia. N. Engl. J. Med..

[16] Thompson WW (2003). Mortality associated with influenza and respiratory syncytial virus in the United States. JAMA.

[17] Fleming DM, Elliot AJ (2005). The impact of influenza on the health and health care utilisation of elderly people. Vaccine.

[18] McElhaney JE, Dutz JP (2008). Better influenza vaccines for older people: what will it take?. J. Infect. Dis..

[19] Derhovanessian E (2010). Hallmark features of immunosenescence are absent in familial longevity. J. Immunol..

[20] Lustgarten J (2009). Cancer, aging and immunotherapy: lessons learned from animal models. Cancer Immunol. Immunother..

[21] Fulop T (2010). Potential role of immunosenescence in cancer development. Ann. N. Y. Acad. Sci..

[22] Wong TN (2015). Role of TP53 mutations in the origin and evolution of therapy-related acute myeloid leukaemia. Nature.

[23] Mullighan CG (2008). Genomic analysis of the clonal origins of relapsed acute lymphoblastic leukemia. Science.

[24] Anderson K (2011). Genetic variegation of clonal architecture and propagating cells in leukaemia. Nature.

[25] Notta F (2011). Evolution of human BCR-ABL1 lymphoblastic leukaemia-initiating cells. Nature.

