# Supplementary material for: Late relapse in acute myeloid leukemia (AML): clonal evolution or therapy-related leukemia?
Source: Blood Cancer J. 2019 Jan 16;9(2):7. doi: 10.1038/s41408-019-0170-3 (PMC6335405; doi:10.1038/s41408-019-0170-3)
Supplement: Supplementary file 1 — Supplementary Appendix [file 41408_2019_170_MOESM1_ESM.pdf]

Methods (Supplementary)

To facilitate the identification of driver mutations, an automatic pipeline was developed to filter and annotate the raw variants called by Mutect and Pindel. First, variants with low quality sequencing data were filtered out. Specifically, variants matching one or more of the following criteria were considered of low quality and therefore filtered out from further analysis: 1) tumor coverage < 15X, 2) tumor allele frequency < 5%, and 3) normal allele frequency >= 1% and 0% for SNVs and INDELs, respectively. Second, only variants which would introduce an obvious protein-coding change were kept for further analysis. Specifically, variant with an ANNOVAR annotation of non-synonymous, stop-gain, stop-loss, splicing, frameshift insertion, frameshift deletion, nonframeshift insertion or nonframeshift deletion was considered to be able to introduce an obvious protein-coding change and were therefore kept for further analysis. Third, common polymorphisms were removed to reduce the load of possible germline contamination due to the absence of matched normal. Specifically, a series of public variant database including the 1000 Genome Database (http://www.1000genomes.org/), ESP6500 Database (http://evs.gs.washington.edu/EVS/), dbSNP ver.129 (http://www.ncbi.nlm.nih.gov/SNP/), and Exome Aggregation Consortium database (http://exac.broadinstitute.org/), were utilized. Variant with a population frequency of 0.14% or more in any of the databases was considered possible germline polymorphism and was therefore removed from further analysis. Finally, a hierarchical classification system was developed to assign confidence level for each remaining variant in order to facilitate the identification of putative driver mutations. Specifically, each variant was classified based on the following hierarchical order and was assigned a confidence level corresponding to its rank in the system: 1) Confirmed somatic mutation based on COSMIC database (version 81), 2) loss-of-function mutation such as splicing, stop-gain, stop-loss and frameshift mutation in tumor suppressor genes, 3) variant which resides in the same position as a confirmed somatic mutation according to the COSMIC database, 4) recurrent variant which resides within three amino acids away from a confirmed somatic mutation according to the COSMIC database and was predicted to be damaging by in-silico function prediction algorithms. The final annotated variant list was then further analyzed by manual inspection in order to identify putative driver mutation.

Supplementary Table 1. Clinical Characteristics of Patients (n=15)

	Diagnosis				Relapse					
UPN	Age, Sex	Karyotype	Induction	CR1 Duration, Y	Karyotype	Salvage#1	Response	CR2 Duration, M	Last Follow up	Reason for Death
1	17M	normal	IA	24.1	normal	CLIA	refractory	n/a	death	Active AML
2	44F	normal	FA	19.1	normal	3+7	CR	62.1 +	alive	n/a
3	34M	t(9;11) (p22;q23)	DCTER	12.1	trisomy 8, del 9q	IA + LY25	CRi	1.6 +	death	Infection
4	66M	del 7q,inv 16,+22	FLAG	9.8	complex	AZA + Vo	refractory	n/a	death	Active AML
5	44M	trisomy 21	DCTER	8.2	normal	IA+Sora	CR	2.8	death	Active AML
6	38F	normal	DCTER	7.5	normal	BID FA	CR	10.5	death	Active AML
7	57M	normal	IA	7.5	normal	SGI-110	CRi	10.8 +	death	TRM
8	58M	normal	IA	7.4	normal	unknown	CR	48.5	death	Active AML
9	75F	normal	IA-IL11	7.1	monosomy 7	AZA	refractory	n/a	death	Active AML
10	37F	del 7q	DNR+AC	6.3	del 5q, del 7q	IA	CR	39.8	death	Active AML
11	60F	normal	IA	6	normal	IA	CR	54.2	death	Active AML
12	67F	normal	Clofa+AC	5.9	normal	Clofa+AC	CRi	66.1 +	alive	n/a
13	66M	normal	Clofa+AC	5.8	trisomy 13	SGN-33A	CR	0.5 +	death	Infection
14	63M	normal	IA	5.6	t(17;20)(q12;p13)	IA	CR	44.4+	death	Unknown
15	60M	normal	IA+SAHA	5.5	normal	AC+BL8040	CR	21.1 +	death	Unknown

UPN, unified patient number; BM, bone marrow; CR1, first complete remission; Y, year; M, male; F, female; CR2, second remission; CRi, CR with incomplete count recovery; M, month; n/a, not applicable; 7+3, daunurobicin, cytarabine; IA, idarubicin, high dose cytarabine; FA, fludarabine, cytarabine (AC); DCTER, daunurobicin, cytarabine, thioguanine, etoposide, and dexamethasone; FLAG, fludarabine, high dose cytarabine, G-CSF; LIPO-DNR, liposomal daunurobicin; Clofa, clofarabine; AC, low dose cytarabine; SAHA, suberoylanilide hydroxamic acid; CLIA, cladribine, idarubicin, cytarabine; AZA, azacitidine; Vo, vorinostat; Sora, Sorafenib; +, reflects ongoing CR at last follow-up;

UPN 9 had chloroma [skin], UPN 11 had chloroma [breast only, BM negative] and relapsed as extramedullary only

Supplementary Table 2. Clinical Characteristics: Baseline vs. Relapse

Characteristics	Baseline N (%)/median [range]	Relapse N (%)/median [range]	P value
Age, year	58 [17-66]	65 [41-82]	0.14
Age ≥65	4 (27)	8 [53]	0.13
WBC, x10^9/L	3.3 [1.1-135]	3.8 [1.1-87]	1
Hg, gr/dl	10.2 [8.5-12.2]	11.2 [7.3-13.2]	1
PLT x10^9/L	129 [12-345]	101 [26-258]	0.7
LDH, U/L	578 [291-3075]	631 [410-3642]	1
T.Bili, mg/dL	0.5 [0.3-0.8]	0.5 [0.2-1.7]	1
Creatinine, mg/dL	1.0 [0.7-1.7]	1.0 [0.5-1.4]	0.9
PB blasts, %	3 [0-98]	0 [0-89]	0.66
BM blasts, %	57 [3-93]	22 [0-86]	0.06
BM Dysplasia	5 (33)	8 [53]	0.57
Cytogenetics			0.12
Favorable	1 (7)	0 (0)	
Intermediate	13 (86)	11 (73)	
Unfavorable	1 (7)	3 (20)	
Unknown	0 (0)	1 (7)	
ORR	15 (100)	12 (80)	0.22
CR	15 (100)	9 (60)	
CRi	0	3 (20)	
CR Duration, year	7.4 [5.9-24]	4.1 [0.1-5.5]	0.01

WBC, white blood cell; Hg, hemoglobin; LDH, lactate dehydrogenase; T. Bili, total bilirubin; PB, peripheral blasts; BM, bone marrow; ORR, overall response rate; CR, complete response
